# Development of machine learning prediction models for postoperative outcomes in adult male circumcision

**DOI:** 10.1186/s12894-026-02072-x

**Published:** 2026-02-10

**Authors:** Leonid Shpaner, Giuseppe Saitta

**Affiliations:** 1 University of California, Los Angeles, Los Angeles, USA; 2 Istituto Clinico Città Studi, Milan, Italy

**Keywords:** Circumcision, Postoperative outcomes, Machine learning, Support vector machines, SHAP, Surgical risk prediction

## Abstract

**Background:**

Male circumcision is among the most commonly performed and clinically endorsed surgical procedures globally, deeply rooted in medical, cultural, and religious traditions. While circumcision confers well-documented health benefits such as reduced infection and inflammation, adult patients often experience variable outcomes related to anatomical variations and comorbidities, emphasizing the importance of optimizing procedural planning.

**Methods:**

The objective of this study was to develop and internally validate prediction models for short-term postoperative complications following adult male circumcision. This retrospective study evaluated the ability of supervised machine learning models (logistic regression [LR], random forest [RF], and support vector machines [SVM]) to predict short-term postoperative complications following adult male circumcision, using procedural and intraoperative variables, including surgical modality (scalpel- and clamp-based (traditional) vs. laser-based), intraoperative blood loss, and operative technique. Data from 194 adult male patients (≥ 18 years) who underwent circumcision between 2023 and 2024 at a single clinical center in Milan, Italy, were analyzed. Models were trained using standardized preprocessing pipelines and evaluated via stratified 10-fold cross-validation using classification metrics, calibration curves, and SHapley Additive exPlanations (SHAP)-based interpretability analysis.

**Results:**

The SVM model demonstrated superior predictive performance, achieving the highest area under the curve of the receiver operating characteristic (AUC ROC) of 0.907, sensitivity of 0.862, average precision of 0.832, and the lowest Brier score of 0.105. SHAP analysis identified intraoperative blood loss and surgical technique as the strongest predictors of postoperative complications.

**Conclusions:**

These findings support the clinical utility of interpretable machine learning models for individualized risk prediction in adult circumcision, guiding tailored preoperative decisions, particularly in high-risk or resource-limited clinical settings. Study strengths include rigorous evaluation and interpretability, while limitations encompass single-center data and the absence of external validation. Therefore, future research should assess generalizability across more diverse surgical populations and healthcare environments.

**Supplementary Information:**

The online version contains supplementary material available at 10.1186/s12894-026-02072-x.

## Background

Though often considered routine, male circumcision in adult patients presents a distinct set of clinical challenges due to comorbidities, anatomical variability, and perioperative factors. Beyond its cultural and historical roots, the procedure carries well-established public health benefits, including reduced risks of infection, inflammation, and certain sexually transmitted diseases [1]. Traditionally performed using scalpel excision and suturing, circumcision has more recently evolved with the adoption of carbon dioxide (CO₂) laser-based techniques, which offer advantages such as improved hemostasis, shorter operative times, and faster recovery [2].

Clinical research, including the work of Leonardi and Saitta [3] has demonstrated reduced intraoperative bleeding, lower infection rates, and improved wound healing with laser-based methods. These findings have driven increasing interest in adopting laser techniques, particularly for adult patients with more complex risk profiles.

Despite these clinical advancements, postoperative complication rates remain non-negligible, and a reliable, data-driven approach to preoperative risk stratification has yet to be established in this surgical context. Predictive modeling using supervised machine learning has shown promise across various surgical domains but has not been widely explored in adult male circumcision.

While the clinical benefits of each technique have been described, few studies have leveraged predictive modeling to assess complication risk based on procedural factors alone. This study addresses that gap by introducing a data-driven framework to forecast short-term postoperative complications and support individualized surgical risk assessment. Although the present approach incorporates intraoperative variables, these features provide mechanistic insight into procedural risk patterns and establish a foundation for future preoperative risk stratification using preoperative predictors.

## Methods

### Reporting guidelines

The study was conducted and reported in accordance with the TRIPOD statement (prediction model development).

### Study population

The aim of this retrospective, single-center study, based on outcomes extracted from existing clinical records without blinding, was to develop and evaluate machine learning models for predicting postoperative complications in adult male circumcision. Machine learning methods were chosen because they can capture complex, nonlinear relationships and interactions among clinical and procedural variables that may not be adequately modeled using traditional statistical techniques. The study was conducted between 2023 and 2024 at a clinical site in Milan, Italy. Of 202 patients initially identified during the study period, 194 adult male patients (≥ 18 years) were included after exclusions based solely on age. All data were fully de-identified before analysis.

### Outcome definition

The primary outcome was defined as a composite binary variable indicating the presence of any clinician-documented postoperative complication, including bleeding, edema, pain, or infection. Patients were coded as positive for the outcome if any one of these events was recorded within one week of the procedure. Complication data were extracted from structured clinical documentation in the patient record. This composite definition was selected to enhance statistical power and account for potential co-occurrence among complication types.

### Data preprocessing and variable transformation

The dataset was cleaned and standardized to ensure consistency and reproducibility. Body mass index (BMI) was recalculated from height and weight measurements (kg/m²) and categorized using standard clinical cutoffs (underweight < 18.5 kg/m², overweight 25.0–29.9 kg/m², obese ≥ 30.0 kg/m²). Mean arterial pressure (MAP) was derived from systolic and diastolic blood pressure values. Variable names were standardized for clarity and uniformity across predictors.

**Fig. 1:**
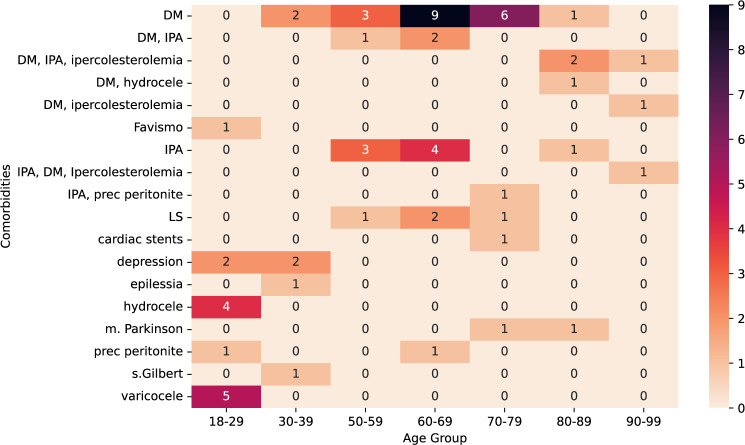
Comorbidities by age group. Frequency of reported comorbidities across age groups. Abbreviations: DM = Diabetes Mellitus, Favismo = Favism, IPA = Arterial Hypertension (Ipertensione Arteriosa), Ipercolesterolemia = Hypercholesterolemia, LS = Lichen Sclerosus, epilessia = Epilepsy, m. Parkinson = Parkinson’s Disease, prec peritonite = History of Peritonitis, s. Gilbert = Gilbert’s Syndrome. DM was the most prevalent

Multiple comorbid conditions were documented in the source data, including ischemic heart disease, hypercholesterolemia, Parkinson’s disease, varicocele, arterial hypertension, and hydrocele. For modeling purposes, only diabetes mellitus was retained due to its higher prevalence and consistent documentation across patients. Among the full cohort (*n* = 194), 64 patients had at least one recorded comorbidity, of whom 30 had diabetes mellitus. Other comorbidities were excluded from model development because of limited sample size or inconsistent documentation. To descriptively characterize the study population, the distribution of recorded comorbidities across age groups was examined (Fig. 1).

### Variables and measurements

Predictors were selected a priori from routinely collected demographic, clinical, procedural, and intraoperative data. The final predictor set included 13 variables: age, body mass index (BMI), surgical technique (traditional vs. laser-based), intraoperative blood loss, operative time, intraoperative heart rate, pulse oximetry, systolic blood pressure, diastolic blood pressure, diabetes status, and BMI category indicators (underweight, overweight, obese). Surgical technique was encoded as a binary indicator (0 = traditional, 1 = laser-based), and categorical predictors (e.g., BMI categories) were converted to binary indicator variables. Predictors with near-zero variance (variance < 0.02) were removed (height, comorbidity indicator, and local anesthesia indicator). After preprocessing, all predictors were numeric and the final modeling dataset contained no missing values. All variables used as candidate predictors, including definitions, units, and measurement timing, are detailed in Supplementary Table S1.

### Model development and evaluation

Three supervised machine learning models were developed to predict postoperative complications: logistic regression, random forest, and support vector machine classifiers. Each model was trained using a consistent preprocessing pipeline that applied MinMax scaling (rescaling numeric features to a uniform range). Evaluation was conducted using stratified 10-fold cross-validation to ensure balanced representation of outcome classes and surgical modalities across all folds. To address class imbalance, all models incorporated balanced class weights. Additional sensitivity analyses using Synthetic Minority Oversampling Technique (SMOTE) and random oversampling were conducted; neither materially changed model rankings. Hyperparameter tuning was performed via grid search across defined parameter ranges, using average precision as the scoring metric for all models. In selecting the best model candidate from each configuration series, the highest average precision score on the out-of-fold samples was used as the determining criterion. For logistic regression, penalty strength was varied; for random forest, the number of estimators, maximum tree depth, and minimum samples per split were tuned; and for SVM, kernel type, cost parameter, and gamma value were optimized. The final selected hyperparameters for each model correspond to the configuration yielding the highest average precision during cross-validation and are reported in Supplementary Table S2. Classification thresholds were optimized using the beta-weighted F-score, which balances precision and recall (sensitivity) based on the relative importance of false negatives. The optimal threshold was selected by maximizing:

$$\:{F}_{\beta\:}=\left(1+{\beta\:}^{2}\right)\cdot\:\frac{\mathrm{P}\mathrm{r}\mathrm{e}\mathrm{c}\mathrm{i}\mathrm{s}\mathrm{i}\mathrm{o}\mathrm{n}\cdot\:\mathrm{R}\mathrm{e}\mathrm{c}\mathrm{a}\mathrm{l}\mathrm{l}}{{\beta\:}^{2}\cdot\:\mathrm{P}\mathrm{r}\mathrm{e}\mathrm{c}\mathrm{i}\mathrm{s}\mathrm{i}\mathrm{o}\mathrm{n}+\mathrm{R}\mathrm{e}\mathrm{c}\mathrm{a}\mathrm{l}\mathrm{l}}$$

with beta values of 1 and 2 used to explore different trade-offs between precision and recall. Model performance was evaluated using sensitivity, specificity, precision, F1-score, average precision, area under the receiver operating characteristic, and Brier score.

Model calibration curves were generated using Platt scaling to assess the alignment between predicted probabilities and observed outcomes.

The SVM determines the optimal decision boundary by solving the following constrained optimization problem:

$$\:\underset{w,b,\{\xi_i\}}{\mathrm{m}\mathrm{i}\mathrm{n}}\:\left(\frac12{\Arrowvert w\Arrowvert}^2+C{\displaystyle \sum_i}\,\xi_i\right)\;\mathrm{s.t.}\;y_i\left(w^{\mathrm{T}}\phi(x_i)+b\right)\ge 1-\xi_i,\;\xi_i\ge 0$$

Here, $y_{i} \in \{-1, +1\}$ denotes the binary class label, $C$ is the regularization parameter, $\phi(x_i)$ is the kernel mapping function, and ${\xi}_{i}$ are slack variables allowing for some misclassification. The radial basis function (RBF) kernel, used to compute similarity between instances, is defined as:

$$K\left(x,{x}^{{\prime\:}}\right)=\:\exp\:\left(-\gamma\:{\| x-{x}^{{\prime\:}}\|}^{2}\right)\:,\quad \gamma\:=\frac{1}{2\sigma^2}$$

This allows the model to project input features into a higher-dimensional space, where a linear separator becomes viable. In the context of this study, such nonlinear separation is critical for capturing subtle clinical distinctions between patients with and without complications.

All modeling was conducted in Python 3.11.11 using the model tuner library (v0.0.34b1) [4] for pipeline construction, hyperparameter tuning, and performance evaluation. Additional dependencies included eda toolkit (v0.0.19) [5], model metrics (v0.0.4a10) [6], numpy (v1.19.5) [7] and scikit-learn (v1.5.1) [8]. Analyses were executed in a Linux-based development environment.

## Results

The final sample ranged from 18 to 93 years of age (median = 34; mean = 43.13, SD = 21.8). Table 1 summarizes baseline demographic and clinical characteristics of the study population, while Supplementary Figure S1 illustrates the inclusion and modeling workflow. Due to the retrospective design and use of a fixed dataset, no a priori power calculation was performed.

**Table 1:** Baseline clinical characteristics by surgical technique

Variable	Traditional Circumcision (*n* = 132)	Laser Circumcision (*n* = 62)	*P*-value
BMI	24.08 (3.03)	24.06 (2.97)	0.9665
Diastolic BP	86.97 (11.12)	74.35 (20.54)	**< 0.001**
Systolic BP	121.82 (11.37)	123.55 (7.26)	0.2126
Heart Rate (bpm)	76.48 (5.96)	74.76 (5.57)	0.0484
Pulse Oximetry (%)	96.70 (1.80)	96.66 (1.76)	0.8741
Intraoperative Blood Loss (ml)	11.59 (17.64)	0.03 (0.52)	**< 0.001**
Surgical Time (min)	28.70 (5.44)	27.06 (5.94)	0.0684
Age	-	-	**< 0.001**
Age 18–29	79 (59.85%)	6 (9.68%)	-
Age 30–39	16 (12.12%)	6 (9.68%)	-
Age 40–49	4 (3.03%)	6 (9.68%)	-
Age 50–59	4 (3.03%)	13 (20.97%)	-
Age 60–69	9 (6.82%)	21 (33.87%)	-
Age 70–79	10 (7.58%)	10 (16.13%)	-
Age 80–89	7 (5.30%)	0 (0.00%)	-
Age 90–99	3 (2.27%)	0 (0.00%)	-
Obese	8 (6.06%)	3 (4.84%)	0.9918
Overweight	34 (25.76%)	19 (30.65%)	0.5894
Diabetes	11 (8.33%)	19 (30.65%)	**< 0.001**
Bleeding, Edema, Pain, or Infection	57 (43.18%)	1 (1.61%)	**< 0.001**

Values are presented as mean (standard deviation) for continuous variables and number (percentage) for categorical variables. The ‘Underweight’ BMI category was evaluated but excluded from Table 1 due to low sample size (*n* = 3) and no significant between-group difference (*p* = 1.0). Bold values indicate statistically significant between-group differences (*p* < 0.001)

Among the 194 included patients, postoperative complications occurred in 58 individuals (29.9%), while 136 patients (70.1%) experienced no complications. Pearson correlations across modeling features indicated moderate correlations between diabetes status and both age (*r* = 0.50) and BMI (*r* = 0.23), supporting the inclusion of diabetes as a standalone comorbidity variable in the model. Distributions of numeric features such as age, BMI, and intraoperative metrics were found to be unimodal and approximately continuous, supporting their treatment as scaled numerical inputs. Surgical time exhibited a primary unimodal distribution with a minor secondary density peak near 36 min.

**Fig. 2:**
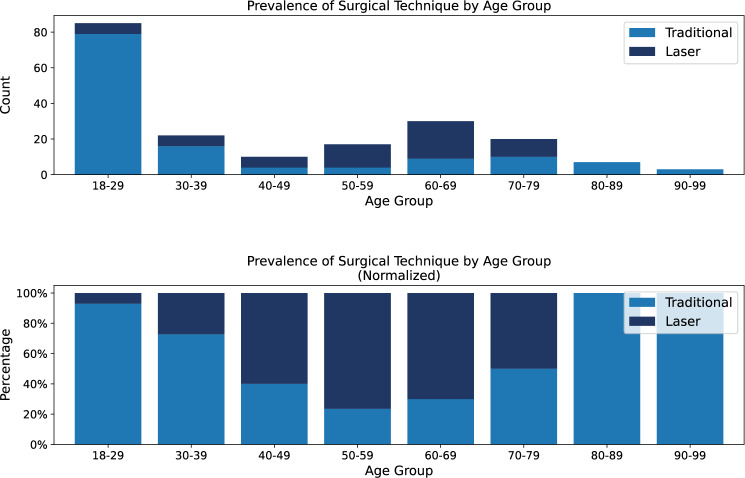
Prevalence of surgical technique by age group. Prevalence of surgical technique by age group, shown as both absolute counts (top) and normalized proportions (bottom). Traditional circumcision was most commonly performed in younger patients, particularly those aged 18-29, whereas laser-based methods predominated in the 50-69 age range. These patterns illustrate age-dependent modality selection and support the inclusion of surgical technique as a modeling feature

While not used as inclusion criteria, data on preoperative antibiotics and anesthesia type were available for review. Cefazolin was the most frequently administered antibiotic, given to patients in the traditional group (*n* = 113) and the laser group (*n* = 62). Use of amoxicillin (*n* = 7), ciprofloxacin (*n* = 8), and gentamicin (*n* = 4) was observed exclusively in the traditional group. Similarly, lidocaine was the predominant anesthetic agent, used in traditional cases (*n* = 128) and laser cases (*n* = 62). Other anesthetics, such as mepivacaine (Carbocaine) (*n* = 4), were used only in the traditional group. Though not included as predictive features, preoperative antibiotic and anesthesia usage patterns were reviewed to characterize perioperative clinical practices that may indirectly influence complication rates. Surgical modality varied notably by age group. Across the full cohort, 68.0% of procedures (*n* = 132) were performed using the traditional method, while 32.0% (*n* = 62) laser-based techniques. Traditional circumcision was predominantly used in younger patients, with 92.9% of procedures among individuals aged 18-29 (*n* = 79 of 85) performed using this method. In contrast, laser-based methods were more common in older age groups, particularly between ages 50-69 (*n* = 47), where traditional technique was used in only 27.7% of cases (*n* = 13). These trends highlight a clear stratification by age and support the inclusion of surgical modality as a covariate in predictive modeling. These trends are further visualized in Fig. 2, which illustrates both the absolute and relative prevalence of surgical modality across age brackets.

All three classifiers (logistic regression, random forest, and SVM) were evaluated using stratified 10-fold cross-validation across key classification metrics. Results are summarized in Table 2.

**Table 2:** Bootstrapped performance metrics (95% confidence intervals) for predictive models of postoperative complications

Metrics	Logistic Regression	Random Forest	Support Vector Machine
Precision/PPV	0.571 (0.469–0.663)	0.706 (0.596–0.810)	0.725 (0.623–0.826)
Average Precision	0.809 (0.704–0.899)	0.737 (0.613–0.875)	0.832 (0.735–0.913)
Sensitivity/Recall	0.897 (0.817–0.966)	0.828 (0.719–0.917)	0.862 (0.765–0.939)
Specificity	0.713 (0.632–0.783)	0.853 (0.786–0.910)	0.860 (0.797–0.915)
F1-Score	0.698 (0.603–0.778)	0.762 (0.667–0.837)	0.787 (0.706–0.857)
AUC ROC	0.900 (0.849–0.943)	0.887 (0.826–0.940)	0.907 (0.855–0.950)
Brier Score	0.137 (0.117–0.160)	0.105 (0.077–0.136)	0.105 (0.077–0.134)

Values are point estimates with 95% confidence intervals shown in parentheses. 95% confidence intervals were estimated using non-parametric bootstrap resampling of patients (*n* = 194; 1,000 resamples) based on out-of-fold predictions. Metrics without parentheses are point estimates only

The SVM consistently outperformed the other models across nearly all evaluation criteria, including precision, recall, specificity, F1-score, AUC-ROC, average precision, and Brier score (Table 2). Model-specific classification thresholds optimized using out-of-fold predictions were applied for performance evaluation (0.238 for SVM, 0.318 for random forest, and 0.439 for logistic regression), as described in the Methods. The SVM achieved the highest area under the receiver operating characteristic (AUC = 0.907), along with the highest F1-score (0.787) and sensitivity (0.862). In addition, the SVM yielded the highest average precision (0.832), indicating a stronger ability to correctly identify true positive cases while minimizing false detections. Specificity (0.860) and precision (0.725) were also higher than those observed in logistic regression and random forest. The Brier score of 0.105, the lowest among the three models, indicated better overall calibration. Random forest also demonstrated strong performance, with an F1-score of 0.762, precision of 0.706, and high specificity of 0.853, although, its sensitivity (0.828) and average precision (0.737) were lower than those of the SVM. Logistic regression, while more interpretable, underperformed in comparison. It produced the lowest precision (0.571), the lowest F1-score (0.698), and relatively low specificity at 0.713. Despite these limitations, logistic regression showed strong sensitivity (0.897) and a competitive AUC of 0.900, indicating effective detection of true positive cases, although its Brier score (0.137) reflected relative to the other two models.

**Fig. 3:**
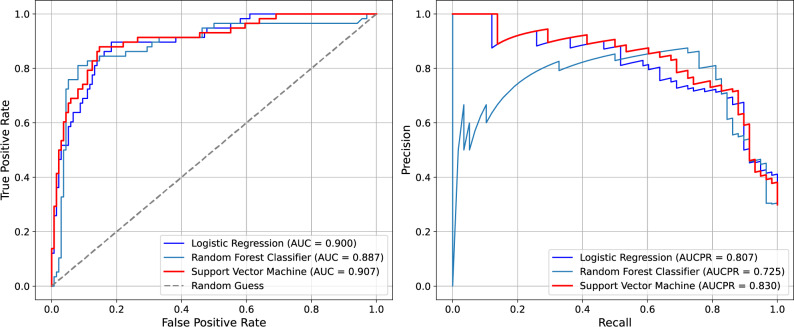
AUROC and PR curves across classifiers. The left panel displays AUROC curves, while the right panel shows PR curves for LR, RF, and SVM. AUROC and Average Precision (AP) values are indicated in the legend. These plots illustrate each model’s ability to distinguish complication risk and maintain predictive confidence under class imbalance

To further illustrate model performance, Fig. 3 displays Area Under the Receiver Operating Characteristic (AUROC) curves for all three classifiers. The SVM demonstrated both strong discrimination and well-calibrated probability estimates, as reflected by the highest AUC (0.907) and average precision (0.832), with predicted performance closely aligned with observed outcomes. Random forest followed closely, while logistic regression showed greater divergence; this is particularly true in settings with class imbalance.

**Fig. 4:**
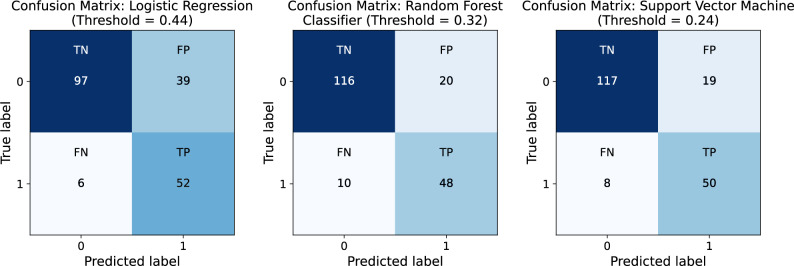
Confusion matrices for Logistic Regression, Random Forest, and Support Vector Machine at optimized thresholds. Each matrix displays the number of true positives (TP), true negatives (TN), false positives (FP), and false negatives (FN) for the respective classifier at its optimized classification threshold. Thresholds were determined via beta-weighted F-score maximization, allowing for a tailored balance between sensitivity and precision

Figure 4 presents the confusion matrices for each classifier at their optimized classification thresholds, determined via *β*-weighted F-score maximization. The SVM demonstrated the most balanced performance, correctly identifying 50 out of 58 complication cases (sensitivity = 0.862) while minimizing false positives (*n* = 19). RF performed similarly, with slightly lower sensitivity (0.828) and 20 false positives. LR, despite achieving the highest sensitivity (0.897), misclassified 39 non-complication cases, demonstrating its lower precision and specificity. These results reinforce the relative advantage of SVMs not only in aggregate performance metrics but also in reliably distinguishing complication risk with minimal overclassification.

### Model interpretability and feature importance

Of the 58 patients who experienced postoperative complications (bleeding, edema, pain, or infection), 57 underwent traditional circumcision and one underwent laser-based circumcision. At the optimized threshold, the SVM correctly identified 50 complication cases, all within the traditional circumcision group. The single complication occurring in the laser group was not predicted by the model likely due to the small number of adverse events in this subgroup, which limits the model’s ability to generalize across underrepresented classes. While this highlights the clinical importance of procedural type and confirms the model’s alignment with observed outcome patterns, it also reveals a specific limitation. Predictive models may fail to detect uncommon complications within subgroups characterized by low complication rates. This observation emphasizes the necessity of thorough error analysis when interpreting model performance, especially in scenarios involving imbalanced classes and influential predictive features. Table 3 cross-tabulates observed and SVM-predicted (radial basis function kernel; C = 100, gamma = auto) complication status by surgical technique, showing that most false-positive predictions (17 of 19) and the majority of false negatives occurred in the traditional circumcision group, while two false positives and one missed complication were observed in the laser group.

**Table 3:** Comparison of observed and predicted complications by surgical technique (SVM model)

Metric	Traditional Circumcision (*n* = 132)	Laser Circumcision (*n* = 62)	Total (*n* = 194)
Observed Complications (n)	57	1	58
SVM Predicted Positive (n)	69	0	69
True Positive (TP)	50	0	50
True Negative (TN)	58	59	117
False Negative (FN)	7	1	8
False Positive (FP)	17	2	19

Complication counts are presented by observed and SVM-predicted outcomes at the optimized threshold (*β* = 1), stratified by surgical technique

To improve interpretability, SHAP values were computed to quantify each feature’s contribution to the predicted risk of postoperative complications produced by the SVM classifier (radial basis function kernel; C = 100, gamma = auto). SHAP provides a model-agnostic approach to estimating feature influence attributing the predicted output to individual features for each case. In this context, it confirmed that intraoperative blood loss and surgical technique were the most influential variables in the model’s decision-making process. This level of transparency supports clinical validation and strengthens the credibility of machine learning predictions in surgical risk modeling [9].

**Fig. 5:**
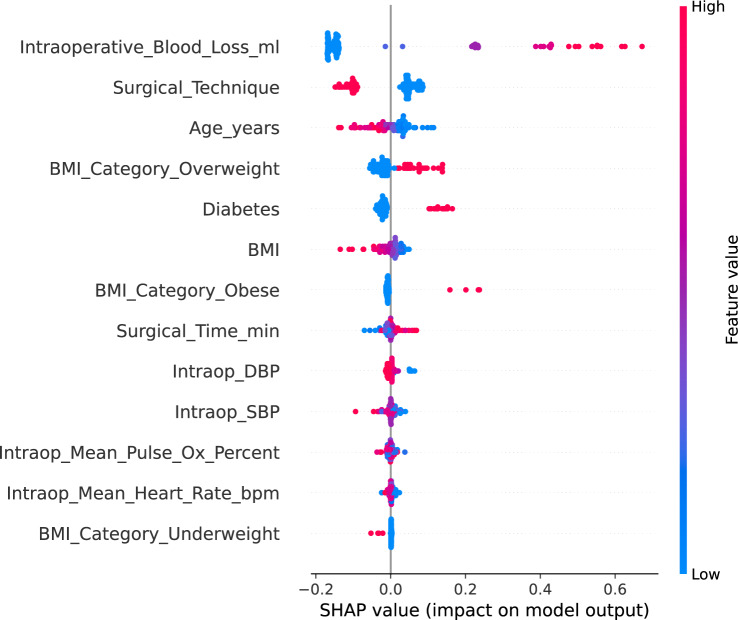
SHAP summary plot showing feature contributions to predicted risk. Each dot represents a single patient. Feature values are color-coded from low (blue) to high (red). SHAP values indicate each feature’s contribution to the predicted risk of postoperative complications, with higher values representing stronger influence on model output. Features ranked higher on the y-axis contributed more to overall predictions across the cohort. For binary variables such as surgical technique and diabetes, the clustering of blue points reflects the larger number of patients in the traditional circumcision and non-diabetic groups, respectively

As shown in Fig. 5, intraoperative blood loss displays a clear monotonic relationship with predicted risk. Patients with higher blood loss consistently exhibited elevated risk scores, while those with minimal blood loss tended to fall on the lower end of the risk spectrum. Similarly, patients undergoing traditional surgical methods were more likely to be classified as high risk compared to those treated with an alternative technique. SHAP analysis revealed that age, diabetes status, and overweight BMI category consistently contributed to higher predicted risk in distinct patient subsets, reflecting their influence within the model’s learned relationships. Notably, SHAP values for diabetes showed relatively consistent positive effects, reinforcing its role as a high-confidence complication risk factor. In contrast, BMI-related variables (e.g., obesity vs. overweight) demonstrated a wider distribution of effects, suggesting that risk is mediated by interactions with other clinical factors rather than BMI alone.

Additionally, both clinical vitals (e.g., diastolic pressure, pulse oximetry) and procedural metrics (e.g., surgical time) exhibited modest but heterogeneous SHAP contributions, highlighting patient-specific physiological variation. This variability demonstrates the model’s capacity to capture individualized risk signals across a range of perioperative inputs. These results not only support the face validity of the model’s reasoning but also offer actionable insights for perioperative planning, enabling more individualized procedural decisions based on risk profiles.

### Use of the prediction model

For an individual patient, the prediction model estimates the probability of experiencing a postoperative complication within 7 days based on preoperative and intraoperative variables, including age, BMI, surgical technique, intraoperative blood loss, operative time, vital signs, and diabetes status. Predicted probabilities were generated from the final trained models, and model-specific probability thresholds were applied to classify patients as high or low risk for postoperative complications. Threshold selection procedures are described in the Methods section.

## Discussion

**Fig. 6:**
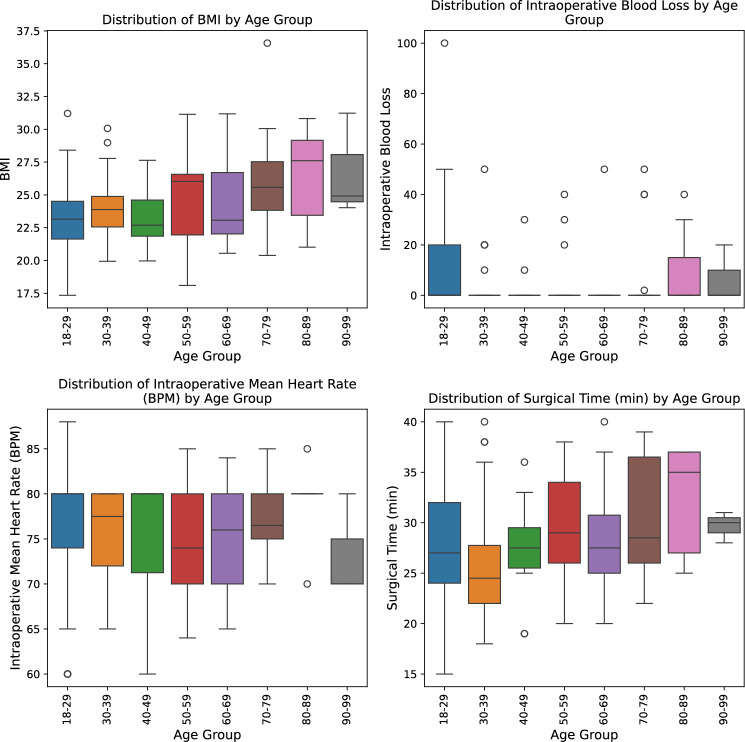
Distribution of intraoperative and procedural variables by age group. Boxplots display BMI, intraoperative blood loss, surgical time, and heart rate across age brackets. These trends contextualize risk heterogeneity and reinforce inclusion of these variables in modeling. These findings not only reinforce the predictive value of comorbidities and intraoperative factors but also motivate a deeper investigation into how specific features influence individual risk predictions, an essential step toward model transparency and clinical trust

These findings align with prior literature indicating that both patient comorbidities and intraoperative variables significantly influence postoperative outcomes in adult circumcision procedures [10]. Conditions such as diabetes and overweight status are known to impair healing and increase the risk of complications. Diabetes has been associated with delayed wound healing and an elevated risk of infection, especially in surgical patients [11]. Intraoperative variables, including blood loss and surgical duration, provide real-time indicators of procedural complexity and physiological stress. Previous research has shown that complication rates in circumcision depend not only on the surgical technique used but also on factors such as anatomical abnormalities and the patient’s overall medical condition, including comorbidities and age [12]. The present work builds on this evidence by quantifying the influence of these factors on predicted complication risk through interpretable machine learning models. By doing so, it offers a transparent and data-driven framework for identifying which clinical variables most strongly contribute to predicted postoperative outcomes. This helps bridge clinical relevance with algorithmic reasoning and supports a more informed approach to postoperative surgical risk assessment. Additional distributions of key intraoperative variables by age group are provided in Fig. 6 to further contextualize these patterns.

These findings not only reinforce the predictive value of comorbidities and intraoperative factors but also motivate a deeper investigation into how specific features influence individual risk predictions, an essential step toward model transparency and clinical trust.

**Fig. 7:**
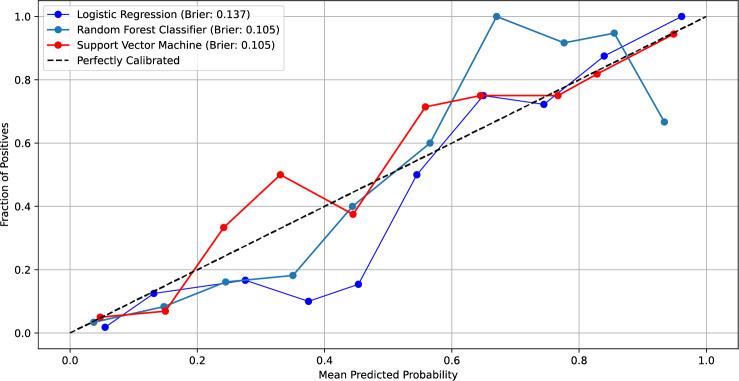
Model calibration curves after Platt scaling. Calibration curves compare predicted probabilities against actual observed event rates. A well-calibrated model aligns closely with the diagonal line representing perfect calibration. Among all models, the SVM demonstrated superior calibration, as reflected by its low Brier score, indicating greater reliability in its probability estimates compared to logistic regression and random forest

In clinical settings, precise calibration is essential when risk estimates guide patient management [13]. Figure 7 shows that the SVM demonstrated the closest alignment with perfect calibration, reinforcing its suitability for real-world decision support in perioperative planning.

Its nonlinear RBF kernel enables flexible decision boundaries that capture complex feature interactions, improving discrimination of patients at elevated risk for postoperative complications.

Taken together, these findings highlight the SVM as the most effective model for predicting short-term postoperative complications following circumcision, particularly when both recall and precision are prioritized. While RF offers a strong alternative with slightly better interpretability, LR may be more appropriate for exploratory or transparency-focused applications rather than high-stakes prediction.

### Limitations

The present study has several limitations that should be acknowledged. Our analysis is based on retrospective data from a single clinical center, which limits generalizability to other populations or surgical settings. In particular, laser circumcision was underrepresented in this cohort compared to its prevalence in broader clinical practice, which may limit the generalizability of findings to institutions where laser-assisted techniques are most commonly used. Moreover, only one postoperative complication was observed among laser circumcision cases, which the SVM did not detect. This finding highlights the model’s limited sensitivity within small subgroups where complications are rare. The relatively modest sample size may restrict the precision of our model’s predictions and the stability of performance metrics across diverse patient cohorts. Given the retrospective nature of data collection, the potential for residual confounding or unmeasured variables influencing postoperative complications cannot be entirely ruled out. Furthermore, the predictive models have not undergone external validation, so future prospective studies are needed to confirm these findings and evaluate generalizability.

## Conclusions

This retrospective study demonstrates that support vector machine classifiers trained on intraoperative and procedural features can effectively predict short-term complications following adult male circumcision. Among evaluated models, the SVM exhibited the highest discriminative ability and best calibration, as reflected in its low Brier score and strong AUC. Post-hoc interpretability analysis using SHAP values aligned with clinical reasoning, identifying intraoperative blood loss and surgical technique as the most influential predictors. The model distinguished between traditional and laser circumcision techniques, identifying a markedly lower complication profile in the laser group. When thoughtfully integrated into surgical decision-making, such models may enable more personalized care and help reduce avoidable complications. Future studies should focus on validating these findings in multi-center cohorts and exploring how such models can be integrated into real-world clinical workflows.

## Supplementary Information

Supplementary Material 1.

## Data Availability

Supplementary materials accompanying this article include detailed hyperparameter configurations (Table S2), additional model diagnostics, and preprocessing specifications. The full analytical codebase used in this study is publicly available at (https://github.com/lshpaner/circ_milan). Due to ethical and institutional restrictions, patient-level data are not publicly available but may be shared in de-identified form upon reasonable request, subject to appropriate approvals. A web-based implementation of the final prediction model is provided as a supplementary resource at the time of publication (https://flask.leonshpaner.com/circumscore/). This tool is intended for illustrative and research purposes and is not required to reproduce the analyses reported in this study.

## Abbreviations

AP: Average Precision
AUC: Area Under the Curve
AUC ROC: area under the curve of the receiver operating characteristic
BMI: Body Mass Index
DM: Diabetes Mellitus
Epilessia: Epilepsy
Favismo: Favism
IPA: Arterial Hypertension (Ipertensione Arteriosa)
Ipercolesterolemia: Hypercholesterolemia
FN: False Negatives
FP: False Positives
IQR: Interquartile Range
LR: Logistic Regression
LS: Lichen Sclerosus
MAP: Mean Arterial Pressure
MinMax: Min–max feature scaling to the [0, 1] range
m. Parkinson: Parkinson’s Disease
PPV: Positive Predictive Value (Precision)
PR: Precision-Recall
prec peritonite: History of Peritonitis
RBF: Radial Basis Function
RF: Random Forest
ROS: Random Oversampling
SD: Standard Deviation
s. Gilbert: Gilbert’s Syndrome
SHAP: SHapley Additive exPlanations
SMOTE: Synthetic Minority Over-sampling Technique
SVM: Support Vector Machine
TN: True Negatives
TP: True Positives
