# Correction: Development of machine learning prediction models for postoperative outcomes in adult male circumcision

**DOI:** 10.1186/s12894-026-02126-0

**Published:** 2026-05-06

**Authors:** Leonid Shpaner, Giuseppe Saitta

**Affiliations:** 1 University of California, Los Angeles, Los Angeles, USA; 2 Istituto Clinico Città Studi, Milan, Italy

**Correction: BMC Urol 26, 54 (2026)**

**https://doi.org/10.1186/s12894-026-02072-x**

In this article [1], Fig. 2 appeared incorrectly and has now been corrected in the original publication. For completeness and transparency, both correct and incorrect versions are displayed below.

The original article has been corrected.

Incorrect Figure

**Fig. 2:**
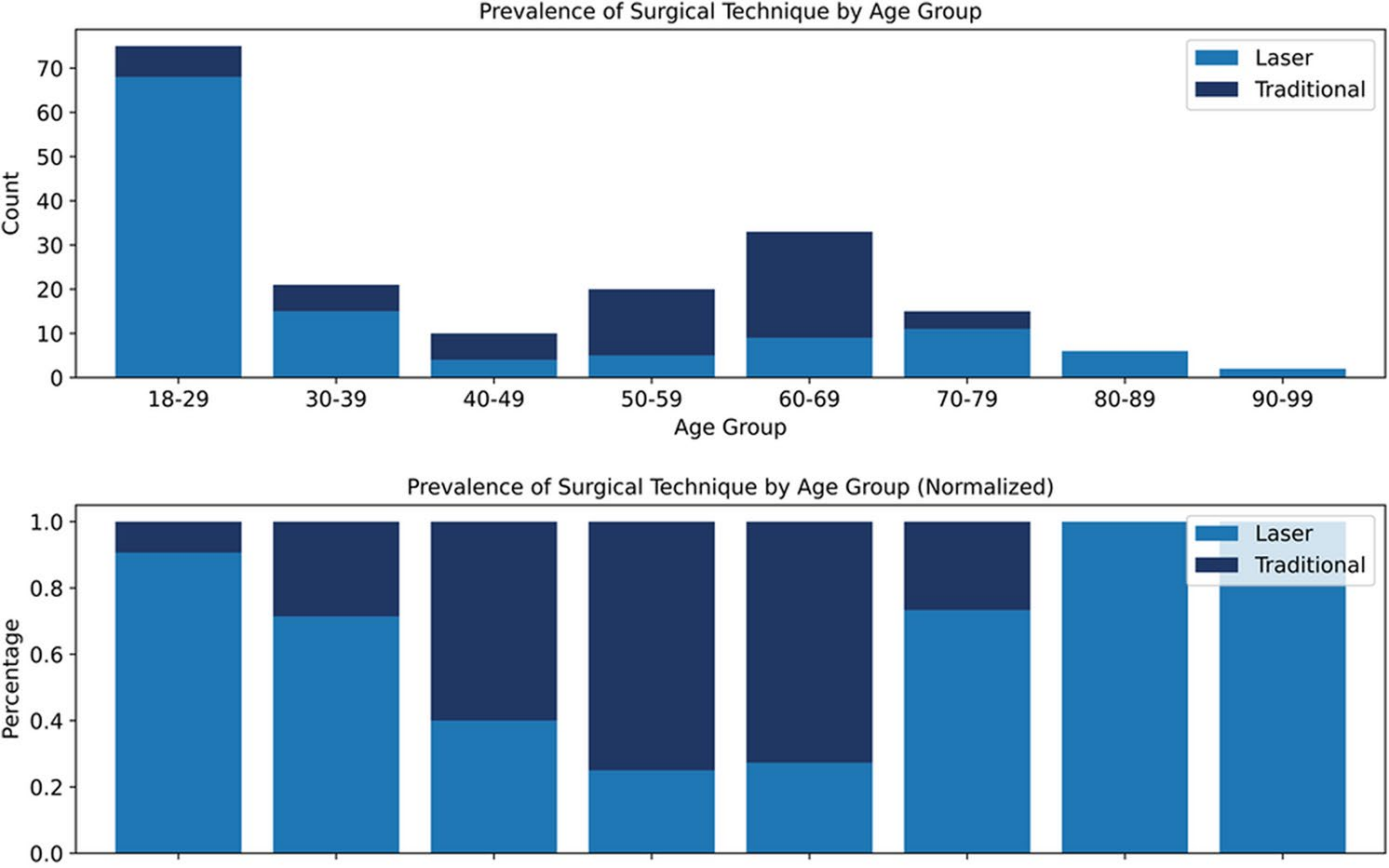
Prevalence of surgical technique by age group. Prevalence of surgical technique by age group, shown as both absolute counts (top) and normalized proportions (bottom). Laser-based circumcision was most commonly performed in younger patients, particularly those aged 18–29, whereas traditional methods predominated in older age groups. These patterns illustrate age-dependent modality selection and support the inclusion of surgical technique as a modeling feature

Correct Figure

**Fig. 2:**
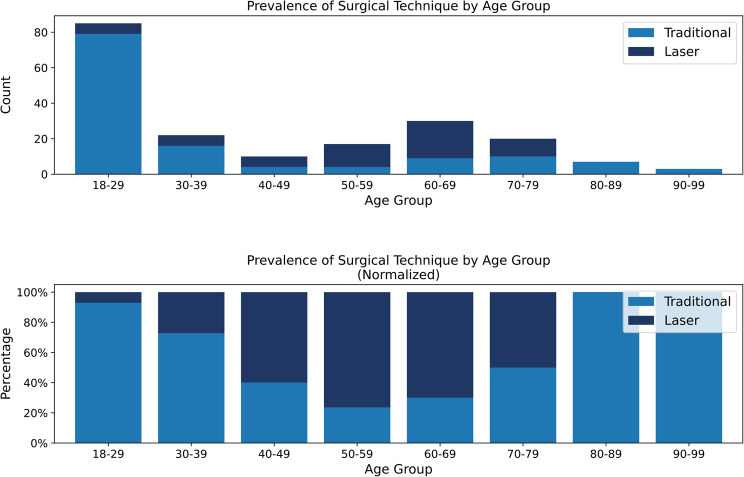
Prevalence of surgical technique by age group. Prevalence of surgical technique by age group, shown as both absolute counts (top) and normalized proportions (bottom). Traditional circumcision was most commonly performed in younger patients, particularly those aged 18-29, whereas laser-based methods predominated in the 50-69 age range. These patterns illustrate age-dependent modality selection and support the inclusion of surgical technique as a modeling feature
