# Supplementary material for: Development of machine learning prediction models for postoperative outcomes in adult male circumcision
Source: BMC Urol. 2026 Feb 10;26:54. doi: 10.1186/s12894-026-02072-x (PMC12951907; doi:10.1186/s12894-026-02072-x)
Supplement: Supplementary file 1 — Supplementary Material 1. [file 12894_2026_2072_MOESM1_ESM.docx]

## **Supplementary Material**

### **Table S1. Predictor variables and measurements used for model development**

| **Predictor** | **Definition** | **Unit / Coding** | **Measurement timing** |
| --- | --- | --- | --- |
| Age | Age of patient at time of surgery | Years (continuous) | Preoperative |
| Body mass index (BMI) | Body mass index calculated as weight/height² | kg/m² (continuous) | Preoperative |
| BMI category: underweight | Indicator for BMI < 18.5 | Binary (0/1) | Preoperative |
| BMI category: overweight | Indicator for BMI 25–29.9 | Binary (0/1) | Preoperative |
| BMI category: obese | Indicator for BMI ≥ 30 | Binary (0/1) | Preoperative |
| Diabetes status | Presence of diabetes mellitus | Binary (0/1) | Preoperative |
| Surgical technique | Type of circumcision performed | Binary (0 = traditional, 1 = laser-based) | Intraoperative |
| Intraoperative blood loss | Estimated blood loss during procedure | mL (continuous) | Intraoperative |
| Operative time | Duration of surgical procedure | Minutes (continuous) | Intraoperative |
| Intraoperative heart rate | Mean heart rate recorded during surgery | Beats per minute | Intraoperative |
| Pulse oximetry | Mean oxygen saturation during surgery | Percentage (%) | Intraoperative |
| Systolic blood pressure | Mean systolic BP during surgery | mmHg | Intraoperative |
| Diastolic blood pressure | Mean diastolic BP during surgery | mmHg | Intraoperative |

### **Table S2. Hyperparameter tuning configurations for evaluated machine learning models**

| **Model** | **Algorithm Type** | **Resampling Method** | **Hyperparameters Tuned** | **Tuning Range/Options** | **Final Selected Hyperparameter Configuration** |
| --- | --- | --- | --- | --- | --- |
| Logistic Regression | Linear Classifier | None, SMOTE, ROS | Penalty (**penalty**) | L2 | Resampling method = SMOTE  penalty = L2  C = 1.0 |
|  |  |  | Inverse Regularization Strength (**C**) | 0.0001, 1.0 |  |
| Random Forest | Ensemble Classifier | None, SMOTE, ROS | Number of Estimators (**n_estimators**) | 10, 50 | Resampling method = SMOTE  n_estimators = 50  max_depth = None  min_samples_split = 5 |
|  |  |  | Maximum Depth (**max_depth**) | None, 10 |  |
|  |  |  | Minimum Samples Split (**min_samples_split**) | 2, 5 |  |
| Support Vector Machines | Kernel-based Classifier | None, SMOTE, ROS | Kernel Type (**kernel**) | Linear, rbf, poly, sigmoid | Resampling method = None  Kernel = rbf  C = 100  gamma = auto |
|  |  |  | Cost Parameter (**C**) | 0.0001 to 100 (log scale) |  |
|  |  |  | Gamma (**gamma**) | 0.001, 0.01, 0.05, 0.1, 0.2, 0.5, scale, auto |  |

**Note:** *SMOTE: Synthetic Minority Oversampling Technique; ROS = Random Oversampling.*


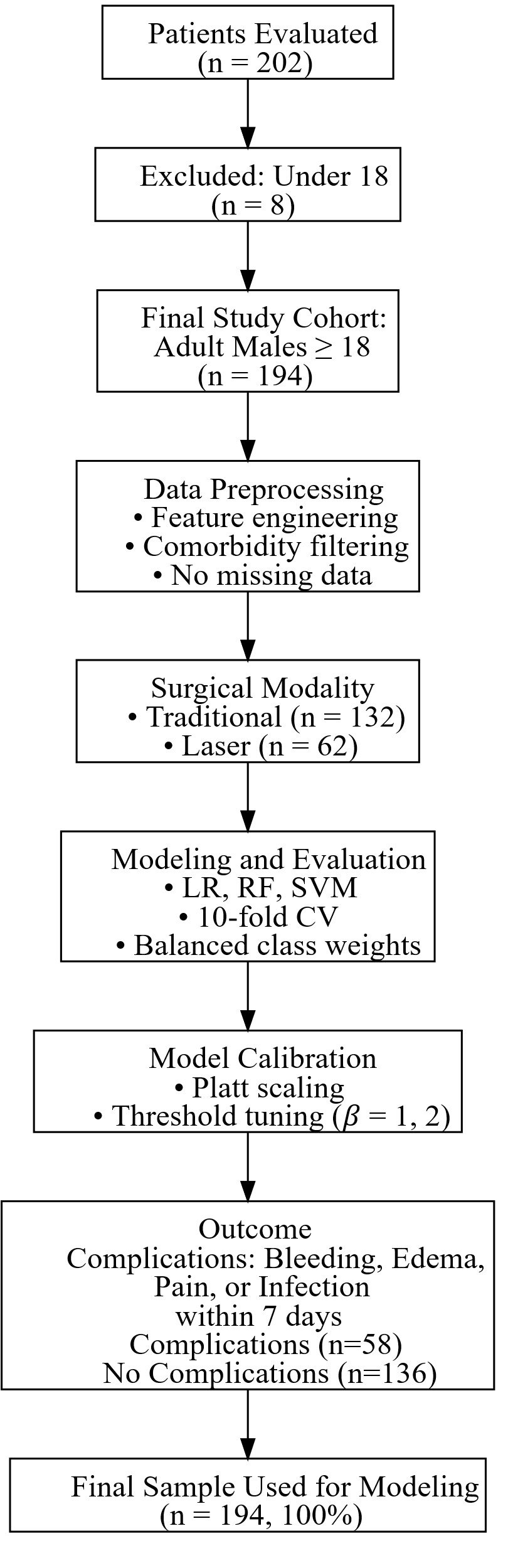


### **Figure S1. Cohort inclusion, preprocessing, and modeling flow:** *STROBE Diagram summarizes patient inclusion, exclusion, data preprocessing, and modeling workflow. Surgical technique was included as a predictive feature and not analyzed as a separate arm. All 194 adult patients were processed through a unified pipeline and included in final model training and evaluation.*
